# Validation of a combined approach-avoidance and conditioned stimulus aversion paradigm for evaluating aversion in chickens

**DOI:** 10.1371/journal.pone.0247674

**Published:** 2021-02-25

**Authors:** Emmarentia W. du Plessis, Ngaio J. Beausoleil, Charlotte F. Bolwell, Kevin J. Stafford

**Affiliations:** School of Veterinary Science, Massey University, Palmerston North, New Zealand; Universidade do Porto Instituto de Biologia Molecular e Celular, PORTUGAL

## Abstract

Understanding animals’ aversion is important to improving their welfare. Aversion is often assessed using an approach-avoidance (AA) test in which animals have to forfeit a reward if they want to avoid an event or environment presented in the same place. However, sometimes the event/environment suspected to be aversive may physically impair the animal’s ability to withdraw from that place (i.e. its ability to express aversion), leading to incorrect interpretations. Combining AA with a Conditioned-Stimulus that predicts the event/environment may overcome this problem by allowing animals to demonstrate aversion without exposure to the stimulus. We aimed to validate this paradigm for testing aversion in chickens. Seven Hyline-Brown chickens were trained to obtain a food reward from a coloured bowl located in the test chamber (TC) of a two-chambered box; the reward was presented in a green bowl with an inactivated air canister or a red bowl with the canister activated to deliver an air puff. Two 5-minute tests were conducted, one with each bowl colour and both with the canister inactivated. All chickens entered TC with the green bowl. With the red bowl, two chickens entered on their first attempt, one fully entered after a partial entry (3/7 fully entered), two made only partial entries and two made no attempts to enter. Chickens spent less time in the TC with the red bowl (median 31s, IQR 7–252) compared to the green bowl (293s, IQR 290–294; p = 0.008). The higher ratio of partial to full entries, failure to enter the TC and less time spent in TC reflected chickens’ aversion to the air puff, signalled by the red bowl. The paradigm allowed chickens to demonstrate aversion without exposure to the aversive stimulus during testing.

## Introduction

Various methods are used to assess aversion in animals. The commonly used approach-avoidance (AA) test relies on creating conflicting motivations in the test subject between staying in an area to access a reward (e.g. food) and leaving that area to avoid a simultaneously administered stimulus or condition suspected to be aversive. A stimulus or condition is deemed to be aversive if the animal leaves the reward in order to avoid it [1–3]. In such paradigms, gross behavioural indicators, such as the time taken to leave the area where the reward is presented, may be used to reflect aversion to the stimulus/condition [4].

Existing methods for evaluating animals’ aversion may be confounded by factors other than the perceived aversiveness of the test condition. Importantly, failure to avoid a stimulus or condition in the AA test may reflect the animal’s physical inability to do so in the test environment rather than a lack of aversion *per se* [5–7]. To illustrate, zebrafish (*Danio rerio*) tested in a standard AA paradigm failed to display avoidance behaviour to the anaesthetic agent tricaine methanesulfonate at the time of exposure and remained in the test location until loss of equilibrium occurred. Those fish, however, showed clear avoidance of the location where the chemical had been encountered when they were subsequently tested in a conditioned place aversion paradigm [7]. Likewise, while broiler chickens exposed to high concentrations of carbon dioxide (CO_2_) in an AA test did not always leave the test environment [2, 5, 8], birds exposed to those CO_2_ concentrations ‘sat down’ much earlier than birds exposed to air [5]. This suggests that their ability to express aversion by leaving the test chamber may have been impaired by the high CO_2_ atmosphere.

Developing a test combining the AA paradigm with conditioned stimulus aversion (CSA) may help address these limitations. During CSA, the test subject is conditioned to associate a certain stimulus with a specific event. In the context of aversion testing, conditioned stimuli are usually visual signals or locations/places [7, 9–11]. Conditioning animals to associate a specific stimulus with the potentially aversive event would allow them to indicate any aversion to the event without actual exposure to it during the test. For example, aversion may be indicated if the animal fails to enter the place where the stimulus is presented, when given the choice to enter or not enter [7]. The reward presented as part of the AA component is used to attract the animal to the place where the potentially aversive event occurs, i.e. to create motivational conflict. It is particularly important to be able to attract the animal back to that place following first exposure to the event during training, in order to facilitate learning of the association between the conditioned stimulus and the event it predicts. Thus, the combined AA-CSA paradigm is also likely to be more successful than a CSA test alone for evaluating aversion.

The aim of this study was to validate a combined AA-CSA paradigm for testing aversion and to identify subtle behavioural indicators of motivational conflict in chickens within this paradigm. The first objective was to demonstrate the presence of gross behavioural indicators of chickens’ aversion to an event known to be aversive. We hypothesised that the chickens would take longer to first enter the test chamber (TC) where a food reward was offered and that fewer chickens would enter the TC when a visual signal associated with an aversive stimulus was presented. We further hypothesised that chickens would spend less time in close proximity to the signal associated with the aversive stimulus than they would with a neutral signal. The second objective was to identify more subtle behaviours expressed by chickens that may reflect their motivational conflict. More subtle behaviours were considered to be those that did not relate simply to entry/avoidance of the TC, and the degree of conflict expressed may provide further information on the aversiveness of the stimulus presented.

## Materials and methods

This study was approved by the Massey University Animal Ethics Committee (MUAEC Protocol 16/30).

### Animals and housing

Twelve 11-week-old Hyline Brown layer pullets were housed indoors as a flock on a deep sawdust litter in a temperature (19 to 21°C) and light controlled fan-ventilated room adjacent to the testing room. The study lasted approximately 6 weeks and was completed before the birds came into lay. An automated 12:12 light-dark regimen (lights on at 7am) was maintained and environmental enrichment provided in the form of perches and nest boxes. Layer pellets (Weston’s Stockfeed, Rangiora, New Zealand) were provided ad libitum during the day except when the group was being trained or tested. Food was removed at the end of each light cycle (7pm) in preparation for training or testing the next day and was returned when all testing/training sessions had been completed for all birds (mid-morning on training days, 4.20pm on the single day of testing). Water was provided ad libitum except during the brief periods of training and testing.

### Experimental apparatus

**Fig 1:**
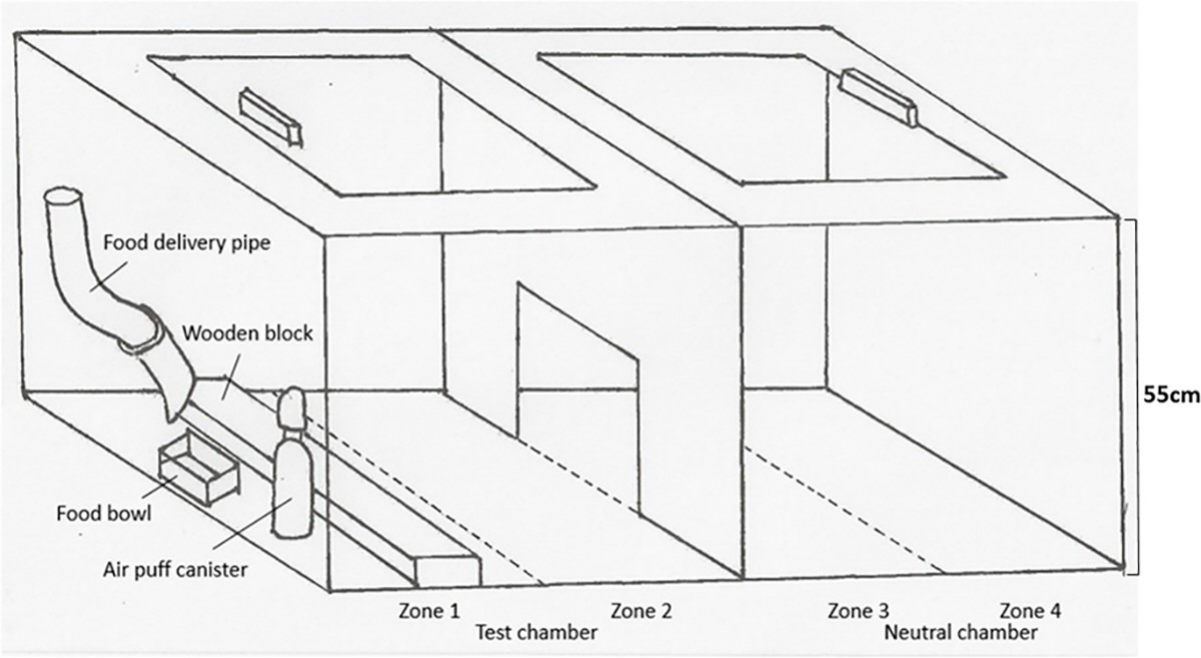
The two-chambered experimental apparatus used to evaluate aversion in chickens.

The experimental apparatus (Fig 1) was positioned in a 20°C temperature-controlled room under two 100W overhead incandescent light bulbs such that illumination was equal on both sides and shadows were eliminated.

The apparatus consisted of a steel box divided into two identical chambers i.e. a test chamber (TC) and a neutral chamber (NC). The interior of each chamber measured 55 cm *L* x 55 cm *W* x 55 cm *H*. The chambers were separated by a white steel partition with a 12 cm *W* x 25 cm *H* pop-hole at floor level allowing free movement between the two chambers. The box was covered by a clear Perspex lid. Each chamber was further divided into 2 zones via lines marked on the lid (Fig 1). The floor of both chambers was covered with a 1.2 cm thick grey non-slip shock-absorbing foam pad.

A flexible PVC tube with an internal diameter of 12 mm penetrated the wall of the test chamber directly opposite the pop hole, with the open end of the tube terminating 5 cm above a bowl (approximately 8cm diameter) to allow remote delivery of a food reward. A rectangular wooden block measuring 55 cm *L* x 10 cm *W* x 2 cm *H* was placed 3 cm in front of the food bowl. The block surface was covered with a grey non-slip shock-absorbent foam pad, and a five-mm white line was painted along each longitudinal edge to aid visualisation of the block on camera.

The aversive stimulus was an audible puff of air delivered via a commercially available automated compressed air canister equipped with a motion detector (Ssscat® spray deterrent, PetSafe, Knoxville, Tennessee). A puff of air has previously been shown to be aversive enough to chickens to elicit a quantifiable behavioural change, but not so aversive as to induce tonic immobility [12–14]. The air canister was located 2 cm to the left of the food bowl. From this position, the device’s sensor detected movement within 12 cm of the food bowl, i.e. within Zone 1 only. In response to movement in this range, it automatically dispensed a brief puff of air delivered through a narrow aperture to create a sudden, moderately loud noise (52 dB measured at 20 cm away, roughly the volume of normal human speech). The angle was set to a height of approximately 10 cm to ensure delivery of the air puff to the chest area in a standing subject, and the face area in a feeding subject. Aversion was assumed to be related to the combination of sudden noise and moving air.

### Test preparation procedure

#### Habituation

The chickens were habituated to the handler, the handling process, and the test environment in a stepwise fashion over three weeks. Chickens were required to meet the performance criterion at the end of each stage to progress to the next stage (Table 1).

**Table 1:** Performance criteria used to determine successful completion of each stage of the habituation and training process.

Stage	Number of chickens at start of stage	Number of chickens that achieved criterion	Performance criterion
**Habituation**			
Handler	12	12	Voluntarily approach and eat dried corn and meal worms from trainer’s hand
Minimal handling	12	10	Show no escape behaviour when placed into, or removed from, a cardboard box
Extensive handling	10	10	Show no escape behaviour when exposed to extensive handling e.g. daily health inspection, leg banding, or drenching
Test environment	10	10	Explore both sides of experimental box for 5 min while separated from flock
**Training**			
Seek reward	10	10	Approach food bowl and wait for food reward while standing on block
Conditioning	10	8	Stand on block for food reward when presented with green bowl (no air puff); avoid standing on block when presented with red bowl (air puff)

#### Training

The trainer was obscured from view during all training procedures to prevent the chickens from forming any association between the trainer and the reward. The chickens were trained in a step-wise fashion over a 1-week period to enter the TC via the pop-hole and stand on the wooden block with both feet to obtain a food reward (freeze-dried meal worms, Topflight, Texas, USA) (Table 1). The purpose of training the birds to stand on the block was to encourage them to stay near the bowl in order to create motivational conflict between receiving the reward and avoiding the air puff. During this phase (“Seek reward”) the food reward was presented in a silver bowl. Initially, the reinforcement schedule for standing on the block was constant (i.e. one worm reward presented immediately for each instance of standing on the block). Once the chickens learned to stand on the block for a food reward, the rewards were offered at random intervals (0–30 s; using numbergenerator.org) during the five-min training session while the chicken was on the block.

During “conditioning” the food reward was delivered into either a green bowl or a red bowl which was visible to the birds from the entrance to the test chamber. The air puff canister was present with both bowl colours but activated only on presentation of the red bowl. Despite some evidence of inherent colour preferences in chickens, specific colour has not been shown to influence avoidance learning in birds [15, 16]. Therefore, the stimulus was conditioned to the red bowl for all chickens.

Conditioning was conducted over nine consecutive days and consisted of a total of 23 training sessions with the green bowl, and five sessions with the red bowl. Starting at 8am each day, each chicken was trained in three sessions with approximately 60 min between sessions (Table 2). More green than red training trials were conducted because chickens initially refused to re-enter the TC when the green bowl was presented after experiencing an air-puff with the red bowl.

**Table 2:** The order in which the green and red bowls were presented each day during the conditioning phase.

Training day	Bowl colour presented			
1	Green	Red	Green	
2	Green	Green	Green	
3	Green	Red	Green	
4	Green	Green	Green	
5	Green	Green	Green	
6	Green	Green	Green	
7	Green	Green	Green	
8	Green	Red	Green	
9	Green	Red	Green	Red

Conditioning was deemed to have occurred when chickens failed to enter the TC within three minutes of being presented with the red bowl. This criterion was set based on the observation that all chickens made a decision to enter the TC or not by three min. Chickens thus appeared to predict the occurrence of the aversive air puff based on the nature of the visual signal within this period. Conditioning training was continued for all chickens, even if they met the performance criterion before day 9. On the last training day, an additional red training session was performed to ensure that adequate conditioning had taken place.

All chickens were highly motivated to obtain dried meal worms at the beginning of habituation. However, reduced motivation was noted after chickens had been exposed to the red bowl and the air puff on the first day of conditioning. From day 2 of conditioning onwards live mealworms (Biosuppliers, New Zealand) were presented to all chickens in all subsequent sessions.

### Testing procedure

Chickens were exposed to two tests, one with the red bowl and one with the green bowl, on the day immediately following the last day of training. All tests were conducted on the same day starting at 8am with chickens having been fasted since 7pm the previous night. The order of testing was randomised and the interval between tests for each chicken was between 20 and 125 min; 5 chickens were presented with the green bowl first and 3 chickens with the red bowl first. The air puff canister was present but not activated in both trials. Between tests, the chickens were returned to their housing and food was not offered till after all testing was complete.

For each test, the chicken was placed into the NC and the behaviour was recorded for five minutes. Live mealworms were presented in the coloured bowl at 30 s intervals during the test period regardless of the location of the chicken. Chickens could see and hear the mealworm fall into the bowl from the NC.

### Data collection

Each chamber of the box was video recorded from above with a separate Sony DCR-SR20 camera (Sony Corporation, Tokyo). Chicken behaviour was scored by a single person from the videos using Behavioural Observation Research Interactive Software (BORIS version 2.998) [17]. The scorer was unaware of the treatments that had been imposed during the conditioning phase and their relationship to bowl colour, i.e. completely blinded. Table 3 shows a detailed ethogram of scored behaviours. Both videos for one chicken had to be excluded from analysis because the air puffer was accidentally left on and was activated during its red bowl test.

**Table 3:** Ethogram of chicken behaviours.

Behaviour	Description	Gross or subtle indicator
**Latencies (s)**		
Time to first full entry into TC	First movement of whole body, including tail, through pop-hole from NC to TC	Gross
Time to first exit from TC	First movement of whole body, including tail, through pop-hole from TC to NC	Gross
**Event behaviours (counts)**		
Look into TC	Head up to hackles moved through pop hole from NC into TC	Subtle
Partial TC entry	Movement of any part of body beyond hackles, but excluding tail, through pop-hole from NC to TC	Subtle
Full TC entry	Movement of whole body, including tail, through pop-hole from NC to TC	Gross
TC exit	Movement of whole body, including tail, through pop-hole from TC to NC	Gross
Failure to enter	Failure to move any body part except head into TC from NC during five-minute test period	Gross
Vocalisation	Vocalisations regardless of pitch, volume or character with number of discrete bouts of vocalisation counted	Subtle
Feather ruffling	Full body feather ruffling including tail	Subtle
Tail wag	Rapid lateral tail movement with one wag counted each time the tail crossed the midline of the body	Subtle
**State behaviours (s)**		
Preening	Time spent preening any body part	Subtle
Time in TC	Time spent with head in TC	Gross
Time in NC	Time spent with head in NC	Gross
Time in Zone 1	Time spent with head in Zone 1	Gross
Time in Zone 2	Time spent with head in Zone 2	Subtle
Time in Zone 3	Time spent with head in Zone 3	Gross
Time in Zone 4	Time spent with head in Zone 4	Gross

TC = test chamber, NC = neutral chamber. Any reference to “head” includes the comb but excludes the hackles.

### Statistical analysis

Latency to enter the TC is presented as a Kaplan-Meier curve of the cumulative probability of the chickens entering the TC over time [18]. This method included data from all chickens, as those that did not enter the TC were classed as censored observations. A log-rank test was used to compare the latency to enter the TC with the red and green bowl. As data were repeated measures and non-normally distributed, Sign tests were used to compare the median time spent in the TC with the red and green bowl, and the median time the chickens spent in each zone (1–4) with each bowl colour. The number of chickens looking into the TC from the NC, partial and/or full entries into the TC and vocalisations were summarised as counts and percentages or median and IQR. All analyses were conducted in Stata (Version 14, StataCorp LP, College Station, Texas, USA), and statistical significance was set at P < 0.05.

## Results

Eight of the 12 chickens successfully completed the habituation and training stages and were used in the testing stage of the study (Table 1). Data for both the red and green bowl for one of these chickens had to be excluded from analysis because the air puffer was accidentally left on and was activated by the bird during its red bowl test (final n = 7 chickens).

### Time to first full entry into the TC

**Fig 2:**
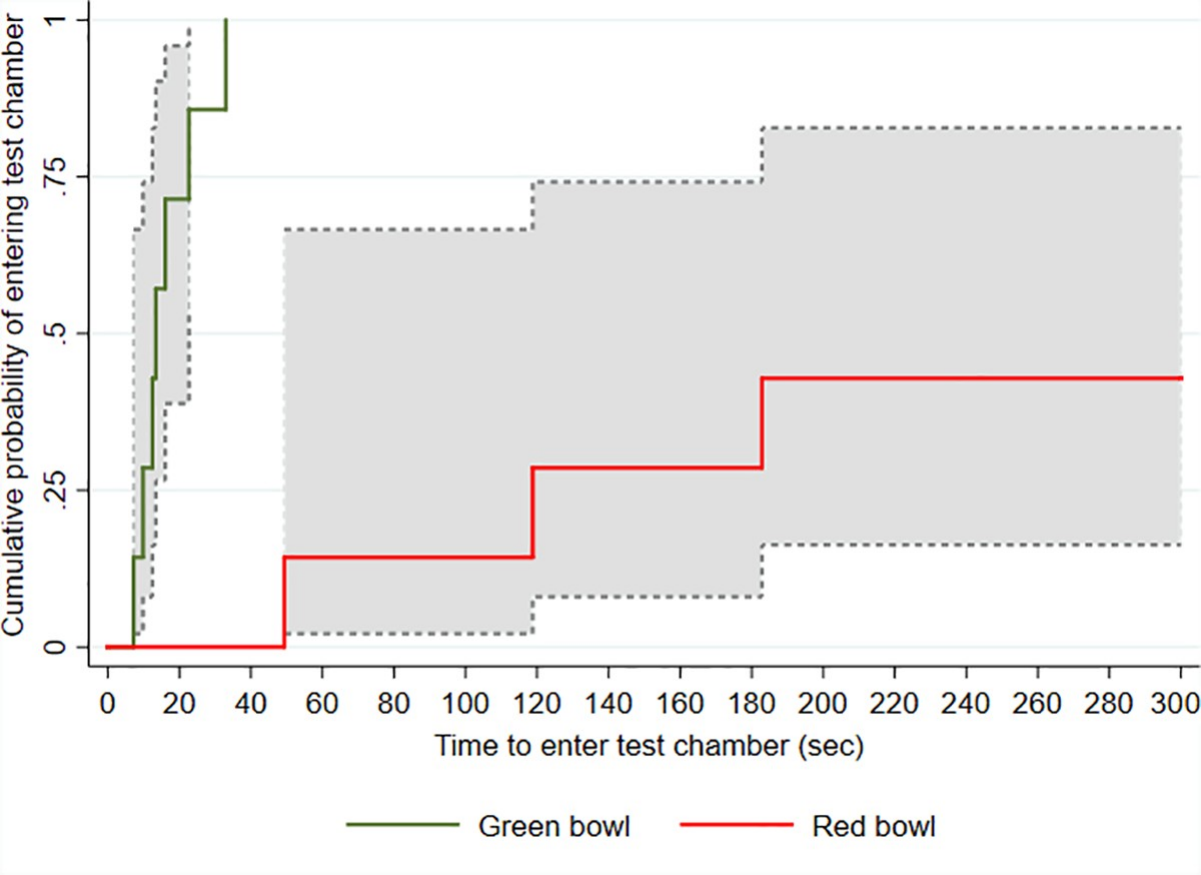
Kaplan-Meier graph of time taken for chickens to enter the test chamber when the red versus the green bowl was present and associated 95% confidence intervals indicated by dotted lines.

The time to first full entry into the TC for all chickens is shown in Table 4. All chickens had entered the TC within 33s when the green bowl was present. Because four chickens did not enter the TC within 300s with the red bowl (right censored), the median time to first full entry could not be estimated. The cumulative probability of entering the TC differed by bowl colour (Log rank test P<0.001; Fig 2). As no chickens left the TC after entry regardless of bowl colour, latency to leave could not be calculated.

**Table 4:** Latency (s) to first full entry into the TC when the green or red bowl was present.

Chicken	Green	Red
1	22.68	49.32
2	13.44	300*
3	12.48	300*
4	9.84	300*
5	33	118.8
6	7.2	300*
7	16.08	182.88

*Did not enter TC within 300s (right censored).

### Time spent in different zones

Chickens spent significantly less time in the TC when the red bowl was present (31s, IQR 7–252) compared to the green bowl (293s, IQR 290–294; p = 0.008). Chickens spent significantly less time in Zone 1 and more time in Zones 2, 3 and 4 with the red bowl than with the green bowl (Table 5).

**Table 5:** Results of sign tests for the median time [interquartile range] spent in Zones 1–4 when the red or green bowl was present.

Zone	Red	Green	p-value
1	0 [0–220]	288 [279–291]	**0.008**
2	28 [7–31]	3 [3–15]	0.06
3	178 [33–207]	5 [4–10]	0.06
4	53 [15–74]	2 [0–2]	**0.008**

P-values refer to differences between red and green bowl presentations for each zone. Bolded values indicate significant differences at P < 0.05.

### Number of chickens entering the TC and expressing other behaviours

Table 6 shows the number of chickens that made full and/or partial entries into the TC. All chickens entered the TC on their first attempt with the green bowl and two chickens with the red bowl. Another chicken fully entered the TC with the red bowl after making a partial entry first. Two chickens failed to enter the TC, even partially, with the red bowl, while a further two chickens made partial entries without subsequently fully entering.

**Table 6:** Entry of chickens into the Treatment Chamber (TC) with the red and green bowl. Data shown as number and percentage of chickens.

Movement into TC	Red Bowl	Green bowl
Failure to enter TC	2 (29)	0 (0)
Partial entry into TC only	2 (29)	0 (0)
Full entry into TC only	2 (29)	7 (100)
Both partial and full entry into TC	1 (14)*	0 (0)

*total >100 due to rounding.

With the green bowl, none of the chickens looked into the TC before entering. With the red bowl, two chickens looked into the TC two or three times and then failed to enter the TC, two chickens looked seven or eight times before partially entering the TC and one chicken looked once before partially and then fully entering the TC. The median number of vocalisation bouts was nine with the red bowl (IQR 5–16, n = 7) and eight with the green bowl (IQR 7–11, n = 7). Only one chicken engaged in preening behaviour which was performed for 80 seconds in the NC on presentation of the red bowl and only after looking into the TC three times. The same chicken did not engage in any preening behaviour with the green bowl and fully entered the TC. One chicken tail-shook three times with the green bowl and not at all with the red bowl. No chickens displayed feather ruffling.

## Discussion

The overall aim of this study was to validate the combined AA-CSA paradigm for testing aversion in chickens by measuring their behavioural responses to a stimulus known to be aversive. Based on the presence of the conditioned stimulus in the TC, chickens demonstrated their aversion by choosing not to enter the TC from the neutral side of the chamber or by taking longer to do so and/or by avoiding Zone 1 where the aversive event occurred during conditioning. This paradigm, thus, allows animals to express aversion without being exposed to potentially confounding treatment effects during testing.

This method could be particularly valuable for evaluating the aversiveness of controlled atmosphere stunning (CAS) methods, such as gases containing high CO_2_ concentrations, because it circumvents the potential risk that some such atmospheres physically impair the animal’s ability to leave the test environment [5, 7]. The paradigm also offers an advantage over the AA test in that it provides an opportunity for decision-making about the test condition prior to entry into the TC, on the basis of the presence or absence of the conditioned stimulus. Thus it may be useful for further assessing the aversiveness of low atmospheric pressure stunning (LAPS) to chickens [19]. LAPS must be applied in a sealed chamber, precluding the use of a standard AA test in which animals express aversion by leaving the test chamber during the test. Using the AA-CSA paradigm, chickens could be conditioned to associate a visual stimulus with exposure to low atmospheric pressure in the sealed chamber. Then, their aversion could be evaluated by their response to the conditioned stimulus without exposure to LAPS during the test itself.

In addition to providing advantages over the AA test, the combined paradigm is more useful than a CSA test alone. Importantly, the reward component of the AA test was required to attract the birds to the place where the aversive stimulus was to be delivered during the conditioning phase and to attract them back to that place after their initial exposure to the air puff. The latter was necessary to allow birds to learn the association between the conditioned stimulus (bowl colour) and the event it predicted (puff or no puff). As evidence, most birds were reluctant to return to the TC after the first air puff and it seems unlikely that they would have done so at all without the food reward to attract them back. The inclusion of an attractive stimulus is particularly important when testing events/environments that develop over time, such as gradual-fill controlled atmospheres used for stunning. The food reward encourages the animal to stay in the environment long enough to experience the relevant gas environment.

The second reason for including a reward (approach) component in this paradigm is that it provides opportunities to explore the relative degree of aversiveness of stimuli of interest. The value of the reward can be manipulated relative to the aversiveness of the unconditioned stimulus. To illustrate, in future we could evaluate the effect of offering a low versus high value food reward on chickens’ willingness to enter the TC when conditioned to expect a 30% CO_2_ environment there. The outcomes could be compared to the birds’ responses to differently valued rewards offered with a 50% CO_2_ environment, providing more detailed information on the difference in aversiveness of the two gas environments. Thus, the combined AA-CSA paradigm offers advantages over either the AA or CSA method of testing aversion alone.

The second objective of this study was to identify subtle behavioural indicators of motivational conflict in chickens. Such behaviours may reflect ambivalence or frustration when an animal simultaneously wants to access a reward and avoid an aversive event or condition [20, 21]. In this study, looking into the TC appeared to be a useful behaviour for recognizing motivational conflict. When the green bowl was present, none of the chickens obviously looked into the TC before deciding to enter. In contrast, when the red bowl was present, five of the seven chickens looked into the TC before either attempting to enter or deciding not to enter at all. These findings are consistent with previous studies, in which vigilance, indicated by changes in chickens’ looking behaviours was associated with the presence of aversive stimuli [12, 22]. Preening, tail shaking and feather ruffling happened too infrequently in this study to be useful for differentiating between treatments. Such behaviours may occur more frequently in response to different aversive stimuli.

Interestingly, the number of attempts to enter the test chamber varied according to bowl colour. While all birds entered the TC fully on their first attempt with the green bowl, three of the seven birds made partial entries with the red bowl; one bird subsequently fully entered while the other two did not. Partial entries could be viewed as vacillation, which often occurs when attraction to a reward is balanced by the desire to avoid a stimulus in the same location [23, 24]. Thus, the ratio of partial to full entries may be a useful indicator of competing motivations in a two-chamber test of aversion. In the current study, it is possible that this ratio was influenced by an inherent preference of some birds for the red-coloured bowl [15, 25], increasing their desire to enter the TC, regardless of the learnt association with the air puff. Evaluating the effects of varying the reward or the aversive stimulus on the expression of these kinds of subtle indicators may provide a more nuanced understanding of the relative strengths of the competing motivations, as opposed to the all-or-nothing outcomes of the standard AA test [2, 3]. This would be useful in the determination of aversion thresholds and dose-response relationships.

All chickens that entered the TC remained there for the duration of the test. This was likely the result of the positioning and activation range of the air puff canister. Chickens were only exposed to the air puff when they entered Zone 1, nearest the food bowl. Since the activation range of the air puff was limited, the red bowl failed to deter three of the chickens from entering the TC as they were able to do so and remain in Zone 2 without being subjected to an air puff. Had the canister been positioned to deliver an air puff immediately upon entry into Zone 2 it is likely that chickens would have spent less time there or would have failed to enter the TC at all. In the case of other potentially aversive treatments such as CAS, the whole chamber would be uniform and a reduction in time spent in the TC and/or refusals to enter may more accurately reflect aversion.

There is a possibility that our exclusion of some birds may have biased the results of the test trials. Two of the 12 birds were excluded before the introduction of the potentially aversive treatment because they failed to habituate to human handling. That is, they were fearful of the handler, which may have impaired their ability to progress to learning the basic feeding task and, subsequently, the association between the bowl colour and the treatment. In addition, continuing to handle them may have compromised their welfare. In future, it would be better to source birds at a younger age and rear them with regular human contact to reduce their fearfulness so that this problem is less likely to occur.

Two additional birds were excluded after apparently failing to demonstrate the ability to discriminate between the red and green bowls. One bird refused to ever go back into the TC after the first exposure to the puffer (in 26 subsequent trials) and thus never had the opportunity to learn the association between bowl colour and treatment. As there was no opportunity for learning, it was appropriate to exclude this bird from testing.

The other bird did re-enter the TC in some of the subsequent 26 training trials, but its behaviour suggested that it failed to discriminate between bowl colours. This may have been because it did not find the air puff aversive and so approached the feeding location regardless of bowl colour. However, in the trial immediately following its first exposure to the air puff with the red bowl, it, like the other birds, refused to re-enter the test chamber, suggesting that at least the initial exposure to the puff was aversive. The air puff we presented was likely to be more aversive than puffs used in previous studies with chickens [e.g. 12] as it was directed at the chicken’s head and was deliberately associated with a loud and sudden noise. Thus, it is most likely that this individual failed to learn the association between signal and treatment. Nonetheless, it should still have been included in the two tests to avoid biasing the results. In addition, it would be valuable in future studies to systematically collect behavioural data during the conditioning trials to better understand the development of aversion learning with stimuli such as the air puff.

A further limitation of our study design was the pairing of the air puff with the red bowl for all birds. There is some evidence of inherent colour preferences in chickens, with red often chosen over green or blue [e.g. 25]. This is not consistent with our observation that chickens avoided the TC when the red bowl was presented, suggesting either that these birds did not prefer red or that they overcame any inherent preference for red in order to avoid the air puff. Similarly, recent work by Arrazola and Torrey [15], employing a conditioned place avoidance paradigm, reported that an initial preference shown by half of the 6 week old broiler breeders for red over blue was quickly over-ridden by their aversion to the associated treatment. This suggests that colour preference was less important than avoidance of the treatment. However, it is important to control for any effects of stimulus colour in future studies, given that colour preference may interact with treatment aversion in unknown ways.

With respect to the outcomes, and despite a limited sample size, the power of the study was sufficient to detect differences between treatments. A retrospective power analysis indicated that there was 85% power to determine a significant difference in the probability of entering the TC for the red and green bowl, should one exist, based on our results for 7 chickens. It is anticipated that larger differences in the behavioural responses would be seen in tests with a more aversive stimulus. However, it should be noted that the diverse behavioural responses to the presence of the red bowl limited statistical comparisons of some of the behaviours recorded in this study.

## Conclusions

This study demonstrates that a combined AA-CSA paradigm is a valid method for testing aversion in chickens and offers some benefits over the use of the standard AA paradigm for evaluating the aversiveness of some conditions. Gross behavioural indicators such as the number of animals willing to enter and the time spent in proximity with the place the aversive event occurred during conditioning were useful for identifying aversion. Subtle behavioural indicators such as the number of attempts to enter and looking into the test chamber before deciding whether the enter may provide additional information on the strength of motivational conflict and thus the animal’s relative aversion to the test condition.

## Supporting information

S1 File (XLSX)
